# Molecular breeding of lignin-degrading brown-rot fungus *Gloeophyllum trabeum* by homologous expression of laccase gene

**DOI:** 10.1186/s13568-015-0173-9

**Published:** 2015-12-22

**Authors:** Misa Arimoto, Kenji Yamagishi, Jianqiao Wang, Kanade Tanaka, Takanori Miyoshi, Ichiro Kamei, Ryuichiro Kondo, Toshio Mori, Hirokazu Kawagishi, Hirofumi Hirai

**Affiliations:** Department of Applied Biological Chemistry, Faculty of Agriculture, Shizuoka University, 836 Ohya, Suruga-ku, Shizuoka, 422-8529 Japan; NARO National Food Research Institute, 2-1-12 Kannondai, Tsukuba, Ibaraki 305-8642 Japan; Integrative Technology Research Institute, Teijin Limited, Iwakuni, 740-8511 Japan; New Business Development Business Unit, Teijin Limited, Tokyo, 100-8585 Japan; Faculty of Agriculture, University of Miyazaki, Miyazaki, 889-2192 Japan; Faculty of Agriculture, Kyushu University, Fukuoka, 812-8581 Japan; Graduate School of Science and Technology, Shizuoka University, Shizuoka, 422-8529 Japan; Research Institute of Green Science and Technology, Shizuoka University, Shizuoka, 422-8529 Japan

**Keywords:** *Gloeophyllum trabeum* KU-41, Laccase gene, Homologous expression, Lignin degradation

## Abstract

The basidiomycete *Gloeophyllum trabeum* KU-41 can degrade Japanese cedar wood efficiently. To construct a strain better suited for biofuel production from Japanese cedar wood, we developed a gene transformation system for *G. trabeum* KU-41 using the hygromycin phosphotransferase-encoding gene (*hpt*) as a marker. The endogenous laccase candidate gene (*Gtlcc3*) was fused with the promoter of the *G. trabeum* glyceraldehyde-3-phosphate dehydrogenase-encoding gene and co-transformed with the *hpt*-bearing pAH marker plasmid. We obtained 44 co-transformants, and identified co-transformant L#61, which showed the highest laccase activity among all the transformants. Moreover, strain L#61 was able to degrade lignin in Japanese cedar wood-containing medium, in contrast to wild-type *G. trabeum* KU-41 and to a typical white-rot fungus *Phanerochaete chrysosporium*. By using strain L#61, direct ethanol production from Japanese cedar wood was improved compared to wild type. To our knowledge, this study is the first report of the molecular breeding of lignin-degrading brown-rot fungus and direct ethanol production from softwoods by co-transformation with laccase overproduction constructs.

**Electronic supplementary material:**

The online version of this article (doi:10.1186/s13568-015-0173-9) contains supplementary material, which is available to authorized users.

## Introduction

*Gloeophyllum trabeum*, along with *Postia placenta*, is a well-characterized brown-rot fungus used as a model laboratory organism. Brown-rot fungi are important recyclers in coniferous forest ecosystems and cause a highly destructive type of wood decay. Thus, elucidating the mechanisms employed by brown-rot fungi in the biodegradation of lignified plant cell walls is very important. A Fenton system is proposed to play an important role in cellulose decomposition by brown-rot fungi (Arantes et al. 2012). This system recently has been receiving increased attention because utilization of this process could facilitate the cost-effective transformation of lignocellulose biomass into biofuel or renewable chemicals (Kerem et al. 1999; Jensen et al. 2001). Therefore, many studies have been conducted to understand the lignocellulose breakdown mechanisms in *G. trabeum*. In addition, decoding of the genomic sequences of brown-rot fungi, including those of *G. trabeum*, has facilitated the identification of key enzymes involved in lignocellulose metabolism (Floudas et al. 2012).

Ethanol is the most widely utilized liquid biofuel alternative to fossil fuels. Cellulosic biomass has been widely regarded as a readily available sugar source to replace starch materials in fermentation. Use of a single microorganism to produce ethanol from lignocellulosic biomass is expected to be especially cost-efficient; such an organism would have to possess the combined abilities to degrade lignin, hydrolyze cellulose, and ferment monosaccharides to ethanol (Lynd et al. 2005). Basidiomycetes play important roles in the carbon cycle as decomposers in forest ecosystems. Recently, we have proposed a new process of unified aerobic delignification and anaerobic saccharification and fermentation of hardwood by a single microorganism, the white-rot fungus *Phlebia* sp. MG-60 (Kamei et al. 2012). In contrast to hardwoods, softwoods such as Japanese cedar are more difficult to degrade by microbial activity (Katagiri et al. 1997). Brown-rot fungi have different mechanisms for the wood degradation with white-rot fungi, which are rapidly depolymerize the cellulose and hemicellulose in wood with modified lignin in the brown residue (Goodell et al. 1997; Hyde and Wood 1997). Brown-rot fungi are accessible to cellulose due to the high xylanase activity (Kim et al. 2014). The accessibility of cellulose increases in *G. trabeum* due to the removal of xylan (Gao et al. 2012). Furthermore, *G. trabeum* has the ability to produce ethanol from sugar under anaerobic or stressed conditions (Rasmussen et al. 2010). Thus, to address this challenge, we are investigating *G. trabeum* KU-41 as a model for the fungal degradation of Japanese cedar wood. Specifically, we hypothesized that it would be possible to develop this strain as a host basidiomycete capable of integrating the delignification, saccharification, and fermentation of softwoods. In this context, the construction of a novel genetic transformation system for *G. trabeum* was expected to permit the construction of strains with improved combinations of these properties. *T. versicolor*, *Pycnoporus coccineus*, *T. hirsute* were used as the laccase-producing wood rot fungi for the control in this study, because these fungi with high laccase production have been widely reported (Bourbonnais et al. 1995; Jaouani et al. 2005; Wu et al. 2011). *Phanerochaete chrysosporium* is a model white-rot fungus, used for comparing the lignin degradation in this study.

In the present study, we developed a novel genetic transformation system for *G. trabeum* KU-41, and demonstrated the molecular breeding of ligninolytic *G. trabeum* KU-41 by the homologous expression of an endogenous gene encoding a putative laccase activity.

## Materials and methods

### Strains and media

*G. trabeum* KU-41 (NBRC 111644) was described previously (Gao et al. 2012). The *G. trabeum* ATCC11539 strain was purchased from the American Type Culture Collection. The typical white-rot basidiomycetes *Trametes versicolor*, *Pycnoporus coccineus*, and *T. hirsuta* were purchased from NITE (National Institute of Technology and Evaluation). The strains used in this study are listed in Additional file 1: Table S1. All strains were maintained on semi-solid CYM medium (Yamagishi et al. 2007) at 28 °C.

### Cloning of genomic DNA fragments encoding *Gtact* and *Gtgpd*

The reagents and protocols for manipulating DNA and RNA (DNA and RNA purification, cDNA synthesis, 3′-RACE, inverse PCR procedure, error-prone PCR, cloning of PCR products) were described previously (Yamagishi et al. 2013). The *G. trabeum* KU-41 genomic DNAs encoding cytosolic actin (*Gtact*) and glyceraldehyde-3-phosphate dehydrogenase (*Gtgpd*) were cloned by a series of PCR procedures (Additional file 1: Figs. S1, S2). The primers that were used are listed in Additional file 1: Table S2. These primers were designed based on nucleotide sequences and intron positions obtained from the DDBJ (http://www.ddbj.nig.ac.jp/). The accession numbers for *Gtact* and *Gtgpd* are AB856051 and AB856282, respectively.

### Construction of transforming plasmids

The construction of transforming plasmids pAH and pGL are depicted in Additional file 1: Figs. S3, S4, S5. The plasmids used for the transformation of *G. trabeum* KU-41 were purified using Quantum Prep Midi kits (Bio-Rad Laboratories KK, Tokyo, Japan).

### Preparation of *G. trabeum* protoplasts

*G. trabeum* protoplasts were prepared by slight modification of the previously described procedure (Yamagishi et al. 2007). Briefly, the KU-41 strain was pre-cultured for 10 days at 28 °C in liquid CYM medium (100 mL/flask in four 500-mL flasks). The mycelium was homogenized with a Waring blender (5000 rpm, 3 min) and cultured (for 4 days at 28 °C) in liquid CYM medium (100 mL/flask in twenty 500-mL flasks). Approximately 5 g of wet mycelium was collected from these cultures and treated with 20 mL of enzyme solution [Lyzing enzymes from Trichoderma (Singma L1412-10G) 2.5 %, Cellulase Onozuka RS (Yakult) 2.5 %/0.5 M MgSO_4_] at 30 °C for 6 h with gentle shaking. Protoplasts were separated from undigested mycelial debris by overlaying onto SorbOsm and centrifuging (4000×*g*) at 4 °C for 20 min. The viability of the resulting protoplasts was estimated as described previously (Yamagishi et al. 2007).

### Genetic transformation procedure

Protoplasts of *G. trabeum* KU-41 (0.8 × 10^8^ cells), marker plasmid pAH (2.5 to 20-µg), co-transformed plasmid pGL (20 µg), and 40 mM CaCl_2_ (final concentration) were combined with SorbOsm to yield a total transformation volume of 1.6 mL. After incubation in ice for 30 min, 50 % PEG solution (1.6 mL) was added, and the combination was gently mixed before being diluted by addition of 20 mL SorbOsm. The transformation mix then was mixed with 150 mL of regeneration medium and poured into 20 Petri dishes (7.5 mL dish^−1^). The composition of the regeneration medium was as described previously (Yamagishi et al. 2007), except that the pH of the medium used in the present study was adjusted to 6.5 (optimal pH for hygromycin) with NaOH prior to use. Following solidification, the plates were incubated at 28 °C for 1 day, and the regenerating protoplasts then overlaid with 7.5 mL/plate of selection medium. (Selection medium consisted of regeneration medium, pH 6.5, supplemented with 40 µg mL^−1^ hygromycin.) Regenerated clones, which appeared in 7–14 days, were picked and subcultured in the individual wells of a 24-well micro-plate containing 1 mL/well of semi-solid CYM medium (pH 6.5) supplemented with 20 µg mL^−1^ hygromycin.

### Amplification of transformed plasmids in regenerated clones

**Fig. 1:**
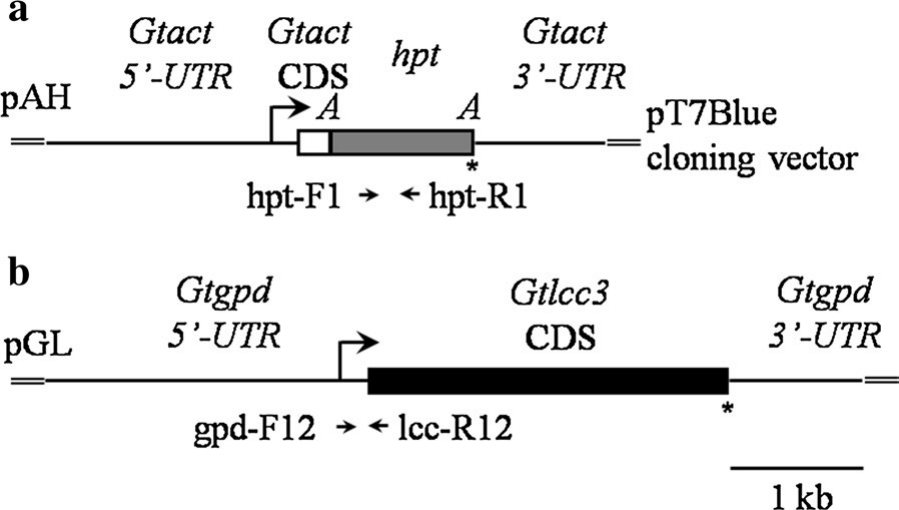
Structures of marker plasmids. **a**, **b** show the overall structures of marker plasmids pAH and pGL, respectively. *Gtact* and *Gtgpd* coding sequence (CDS) indicate *Gloeophyllum trabeum* KU-41 genomic DNA, including introns. The indicated constructs were cloned into the TA-cloning site of cloning vector pT7Blue. The *arrows* indicate the location of primers used for direct PCR. *A* *Asc*I; *asterisk* stop codon

The direct PCR procedure for each regenerated clone was described previously (Yamagishi et al. 2013). Forward primers (hph-F1 and gpd-F12) and reverse primers (hph-R1 and lcc3-R1) were used to amplify the transformed plasmids pAH and pGL, respectively. The locations of the primers within the respective plasmids are depicted in Fig. 1.

### Measurement of laccase activities of clones co-transformed with pGL

Initial screening was performed as follows. For each transformant harboring pGL (44 clones total), two 5-mm^2^ pieces of mycelium were used to inoculate Kirk liquid medium (Tien and Kirk 1988) (2 mL/well in 12-well multititer plate), and plates were grown at 28 °C for 5 days. The laccase activity of each clone (each well) was measured using 3-ethylbenzothiazoline-6-sulfonic acid (ABTS) as the substrate (Nagai et al. 2002). Eleven clones showing appreciable laccase activity were further characterized for laccase activity. For each clone, three flasks were generated as follows: five 5-mm^2^ pieces of mycelium were used to inoculate Kirk liquid medium (10 mL/flask in 100-mL flask), and cultures were grown at 28 °C for 5 days. The laccase activity of each clone was calculated as the mean ± standard deviation of the values from each trio of cultures. The laccase activity was also detected as zymogram. Extracellular proteins were separated by native polyacrylamide gel electrophoresis (PAGE), then visualized as zymogram by laccase active staining with 2,6-dimethoxyphenol.

### Assessment of genetic stability of clones co-transformed with pGL

Transformed clone L#61 was subcultured on semi-solid CYM medium without hygromycin until the border of colony reached the edge of the Petri dish (90 × 15 mm). A piece of mycelium was punched out from the peripheral zone and subcultured on a new plate of semi-solid CYM medium without hygromycin. This process was repeated for a total of five times, and the sequential passages were denoted as generations 1–5. After subculturing, generations 1 and 5 of transformant L#61 were cultured in Kirk liquid medium (10-mL/flask in three 100-mL flasks) and their laccase activities were measured as described above. The laccase activity of each of the two tested generations was calculated as the mean ± standard deviation of the values from each trio of cultures.

### Determination of ligninolytic properties and ethanol production from wood

Erlenmeyer flasks (50-mL) containing 0.5 g extractive-free Japanese cedar wood meal (80–100 mesh) and 1.25 mL distilled water each were inoculated with mycelial discs of wild-type *G. trabeum* KU-41(WT) or transformant L#61 and the resulting cultures were incubated at 30 °C for 28 days. Weight loss, Klason lignin content, and acid-soluble lignin content in the fungal-treated wood meal were determined after the incubation period, as described previously (Hirai et al. 1994). Total lignin content was calculated as sum of Klason lignin and acid-soluble lignin contents.

Following the 28-day incubation, each wood meal culture was supplemented by the addition of 10 mL of basal liquid medium without saccharides (10 g L^−1^ yeast extract, 10 g L^−1^ KH_2_SO_4_, 2 g L^−1^ (NH_4_)_2_SO_4_, and 0.5 g L^−1^ MgSO_4_-7H_2_O, pH 4.5) and then homogenized using a Polytron PT1200E. The cultures were further incubated with shaking (150 rpm) at 30 °C for 3, 6, 9, and 12 days in semi-aerobic conditions (obtained by sealing each culture flask with a silicon plug stopper). After fermentation, the wood meal cultures were centrifuged (10,000×*g*, 5 min). Ethanol levels in the resulting supernatants were measured by high-performance liquid chromatography (HPLC) using a Shodex SH1821 column (8.0 mm × 300 mm, Showa Denko K.K., Tokyo, Japan) at 75 °C with 0.5 mM H_2_SO_4_ as the mobile phase at a flow rate of 0.6 mL min^−1^. Detection was performed using an online Refractive Index Detector.

## Results

### Construction of a genetic transformation system for KU-41

**Fig. 2:**
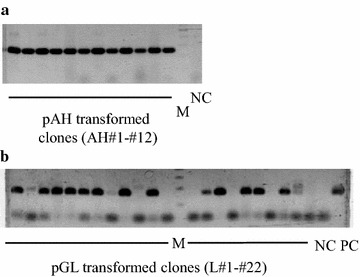
Direct PCR analysis of genomic DNA recovered from individual transformants. Supernatants of homogenized mycelium of the transformants were used in PCR amplification with primer pair hptF1-hptR1 (**a**) or gpdF12-lccR12 (**b**). “M” indicates a 100 bp ladder size marker. “NC” indicates negative control (reactions with no PCR template). “PC” indicates positive control using pGL as PCR template. The PCR products were electrophoresed in 2 % agar and stained with SYBR Green (Takara)

We varied experimental conditions to investigate the appropriate conditions for preparing protoplasts of our strain. A sufficient number of protoplasts (0.2 × 10^8^ protoplasts per gram of wet mycelium, 4 % cell viability) were obtained from the 4-day mycelial cultures. The marker plasmid pAH was constructed to express the hygromycin resistance-encoding gene (*hpt*) under the control of a constitutive promoter (for a gene encoding a cytosolic actin) (Fig. 1a). Genetic transformation of KU-41 protoplasts with 10 µg of the marker plasmid pAH yielded 26–35 transformants; no clones were obtained in the absence of pAH (Table 1). Direct PCR of clones transformed with pAH confirmed that all hygromycin-resistant clones contained the *hpt* gene (Fig. 2a).

**Table 1:** Transformed clones regenerated in uracil-free regenerating medium

Transformed plasmids		Exp. 1	Exp. 2
pAH	20 μg	75	77
pAH	10 μg	26	35
pAH	5 μg	20	24
pAH	2.5 μg	10	17
pAH + pGL	10 + 30 μg	100	106
Control	–	0	0

Transformation experiments were performed twice (experiment 1 and experiment 2)

*Exp* experiment

### Forced expression of the endogenous gene *Gtlcc3*

**Fig. 3:**
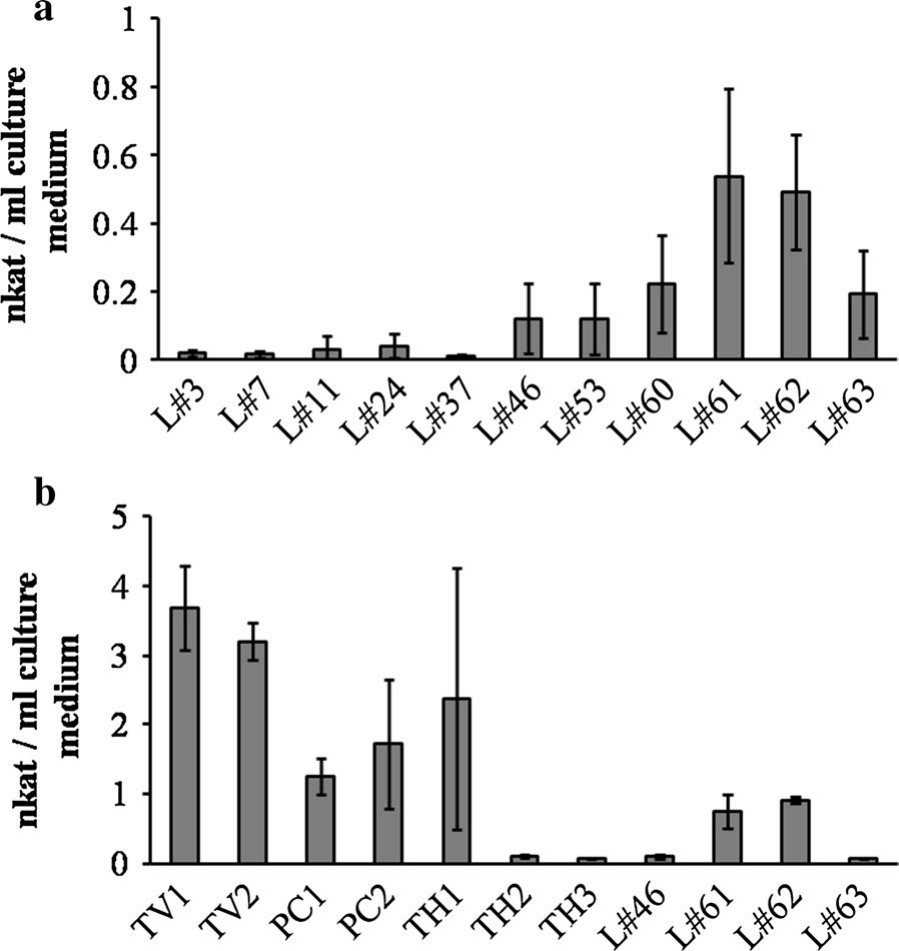
Laccase activities of co-transformants. **a** Laccase activities produced in liquid culture medium by individual transformants. **b** Comparison of laccase producing abilities between the co-transformants and typical laccase producing white-rot basidiomycetes. TV1: *Trametes versicolor* NBRC4937, TV2: *T. versicolor* NBRC30340, PC1: *Pycnoporus coccineus* NBRC6489, PC2: *P. coccineus* NBRC9495, TH1: *Trametes hirsuta* NBRC7038, TH2: *T. hirsuta* NBRC4917, TH3: *T. hirsuta* NBRC6477. All values are presented as mean ± standard deviation (n = 3)

To confirm transgene expression in KU-41, the co-transforming plasmid pGL was constructed (Fig. 1b). pGL was designed to express an endogenous (*G*. *trabeum*) laccase candidate gene (*Gtlcc3*) under control of the *Gtgpd* promoter (Additional file 1: Figs. S4, S5). [*Gtlcc3* represents one of 4 putative laccase genes (*Gtlcc1*–*4*) identified in the *G*. *trabeum* genome database (http://genome.jgi-psf.org/Glotr1_1/Glotr1_1.home.html; protein ID: 127593)]. A total of 72 hygromycin-resistant clones were characterized following co-transformation with pAH and pGL; direct PCR revealed that 44 of the 72 clones harbored pGL (Fig. 2b; Table 1). All 72 clones were inoculated on semi-solid medium containing ABTS. After 2 days, green haloes were visible around 30 of the 44 pGL-positive clones. Haloes were not observed around any of the negative control strains, including the KU-41 parent; 17 hygromycin-resistant strains recovered following transformation with pAH alone; and the separate *G*. *trabeum* ATCC 11539 strain. The production of laccase in Kirk liquid medium also was evaluated (Fig. 3). Co-transformant L#61 exhibited 0.73 nkat mL^−1^ laccase activity. Although the laccase activity of co-transformant L#61 was lower than that of typical white-rot fungus *T. versicolor*, the activity was similar to that of *P. coccineus* (Fig. 3b).

**Fig. 4:**
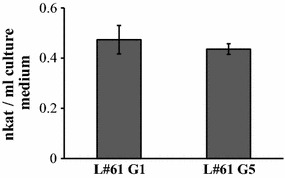
Assessment of genetic stability of transformants in the absence of selective pressure. G1 and G5 indicate generations 1 and 5, respectively. All values are presented as mean ± standard deviation (n = 3)

Co-transformant L#61 was repeatedly subcultured on semi-solid CYM medium without hygromycin to evaluate the genetic stability of the laccase construct in the clones co-transformed with pAH and pGL. Laccase production by the L#61 co-transformant did not appear to decrease even after 5 cycles of subculturing on hygromycin-free semisolid medium (Fig. 4; Additional file 1: Fig. S6).

### Degradation of lignin in Japanese cedar wood meal by co-transformant L#61

**Fig. 5:**
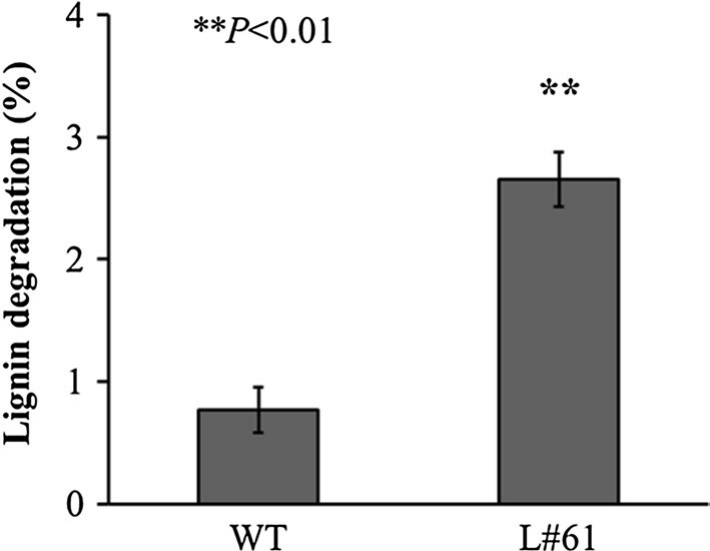
Lignin degradation rate in Japanese cedar wood meals by the parent *G. trabeum* KU-41 (WT) and the co-transformant L#61. All values are presented as mean ± standard deviation (n = 3). *Asterisks* indicate values that were determined by the Student’s t test to be significantly different compared to wild type (**P < 0.01)

We tested whether the L#61 co-transformant was capable of degrading lignin in Japanese cedar wood meal. The co-transformant L#61 exhibited significantly elevated ligninolytic activity (2.7 %) compared to the parent (0.8 %), as shown in Fig. 5. L#61 indicates values that were determined by the student’s *t* test to be significantly different compared to wild type. In a parallel assay, the white-rot fungus *P. chrysosporium* also hardly degraded Japanese cedar wood meal (data not shown).

**Fig. 6:**
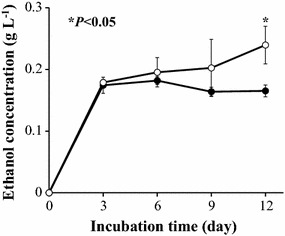
Ethanol production from Japanese cedar wood meal after 28 days of pre-treatment with the parent *G. trabeum* KU-41 (*closed circles*) or the co-transformant L#61 (*open circles*). All values are presented as mean ± standard deviation (n = 3). *Asterisks* indicate values that were determined by the Student’s t test to be significantly different compared to the parent (*P < 0.05)

After a 28-day pretreatment period, the WT or transformant L#61 wood meal cultures were transferred to a basal liquid medium and incubated under semi-aerobic conditions to assess ethanol production. After 12 days of semi-aerobic incubation, co-transformant L#61 exhibited a significant increase (of 45 %) in ethanol production compared to WT (Fig. 6).

## Discussion

In the present study, we investigated the appropriate conditions for preparing protoplasts of the KU-41 strain, because preparation of a sufficient number of protoplasts is essential for construction of a genetic transformation system. Among various parameters (e.g., the age of the mycelium, concentration of the osmotic stabilizer in the enzyme solution, and the composition of the regeneration medium), mycelial age showed the strongest effects on protoplast isolation (data not shown). Older mycelium produced a viscous substance that hindered the enzyme treatment. In addition, because some reports have indicated that native introns (i.e., introns derived from the host species) were essential for the expression of exogenous genes in some basidiomycetes (Lugones et al. 1999; Ma et al. 2001; Burns et al. 2005; Yamazaki et al. 2006; Yamagishi et al. 2013), the first and second introns were retained as part of the *actin* promoter in pAH. However, the necessity of the introns was not confirmed in this study. Genetic transformation of KU-41 protoplasts with the marker plasmid pAH was successful, thus demonstrating our construction of a genetic transformation system for the KU-41 strain of the brown-rot fungus *G. trabeum*. The endogenous laccase candidate gene was fused with the *G. trabeumgpd* promoter and co-transformed with marker plasmid pAH. We obtained 44 co-transformants, and identified co-transformant L#61, which showed the highest laccase activity among all of the screened transformants. Our results indicated that forced expression of the introduced endogenous gene was successful and confirmed that *Gtlcc3* indeed encodes a laccase activity. Although the laccase activity of co-transformant L#61 is lower than that of typical white-rot fungus *T. versicolor*, the activity was similar to that of *P. coccineus*. Transformants with elevated laccase production might be generated by modifying the pGL promoter region and/or by screening among a larger number of co-transformants. Given that pGL does not contain a known origin of replication for *G*. *trabeum* and that the transformed plasmids were stably retained in the absence of selective pressure, we infer that the transformed plasmids were integrated into the chromosomes of the transformants.

Our primary concern was whether the co-transformant L#61 could degrade lignin; the parent *G. trabeum*, like all brown-rot fungi, does not exhibit appreciable lignin degradation activity (Gao et al. 2012). We assayed the ability of co-transformant L#61 to degrade lignin using wood meal derived from Japanese cedar. Laccase mediates one-electron oxidation of phenolic substrates to form many degradation products via various pathways, but the enzyme can not oxidize non-phenolic substrate (Kawai et al. 1989). Thus, co-transformant L#61 showed low delignification activity, because this fungus does not have the delignification systems for non-phenolic structure like white-rot fungi. Additionally, hardwood tree species contain both guaiacyl and syringyl lignin but softwood tree species contain only guaiacyl lignin. Although white rot fungi preferably degrade syringyl units (Dong et al. 2013), they are not contained in Japanese cedar wood (a softwood). Thus, it is suggested that *P. chrysosporium* hardly degraded Japanese cedar wood meal. From the above, it is estimated that co-transformant L#61 was only able to oxidize phenolic residues that were presented at surface of lignin molecules in Japanese cedar wood.

Our results indicated that KU-41 was invested with ligninolytic activity upon the expression of *Gtlcc3*; delignification improved the direct yield of ethanol following fermentation of the wood meal. Autoclave sterilization, which could cause an effect similar to steam explosion pretreatment frequently used as pre-treatment for lignocellulosic substrates, was performed before fungal inoculation. Therefore, it is likely that the wood structure was changed into the structure that has better accessibility for fungal hydrolases to cellulose by the sterilization. In addition, due to relatively high xylanase activity, *G. trabeum* could make wood to accessible structure to cellulose. (Kim et al. 2014). From these reason, *G. trabeum* wild type might have been able to hydrolyze cellulose effectively and produce ethanol, in this experiment. Moreover, it is generally considered that both lignin and hemicellulose limit the accessibility of cellulase. And hemicellulose and lignin are chemically bind. Therefore, we estimated that recombinant laccase assists the hemicellulose removal due to polysaccharide hydrolases as a result of lignin surface oxidation, and that the improvement of lignin and hemicellulose removal leads to higher ethanol productivity of co-transformant L#61.

In conclusion, the homologous overexpression of a putative laccase-encoding gene provided *G*. *trabeum* with ligninolytic activity against Japanese cedar wood. To our knowledge, this is the first report that the molecular construction of a lignin-degrading brown-rot fungus. We expect that our transformation system and the resulting strain constructs will facilitate molecular analysis of the lignocellulose biodegrading system of *G. trabeum* and other brown-rot fungi.

## Additional file

10.1186/s13568-015-0173-9 List of strains used in this study. **Table S2.** Oligonucleotides used as primers in this study. **Fig. S1.** Procedure for cloning *Gloeophyllum trabeum* actin-encoding genomic DNA. **Fig. S2.** Procedure for cloning *Gloeophyllum trabeum* glyceraldehyde-3-phosphate dehydrogenase-encoding genomic DNA. **Fig. S3.** Construction of marker plasmid pAH. **Fig. S4.** Addition of an AscI site in pGtgpd. **Fig. S5.** Construction of pGL. **Fig. S6.** Zymogram activity of the purified laccase enzyme with native PAGE.
