# Supplementary material for: Molecular breeding of lignin-degrading brown-rot fungus Gloeophyllum trabeum by homologous expression of laccase gene
Source: AMB Express. 2015 Dec 22;5:81. doi: 10.1186/s13568-015-0173-9 (PMC4688280; doi:10.1186/s13568-015-0173-9)
Supplement: Supplementary file 1 — 10.1186/s13568-015-0173-9 List of strains used in this study. Table S2. Oligonucleotides used as primers in this study. Fig. S1. Procedure for cloning Gloeophyllum trabeum actin-encoding genomic DNA. Fig. S2. Procedure for cloning Gloeophyllum trabeum glyceraldehyde-3-phosphate dehydrogenase-encoding genomic DNA. Fig. S3. Construction of marker plasmid pAH. Fig. S4. Addition of an AscI site in pGtgpd. Fig. S5. Construction of pGL. Fig. S6. Zymogram activity of the purified laccase enzyme with native PAGE. [file 13568_2015_173_MOESM1_ESM.docx]

Supplementary material

Table S1 List of strains used in this study

| Species | strain | Abbreviations in this study |
| --- | --- | --- |
| *Gloeophyllum trabeum* | KU-41 |  |
| *G. trabeum* | ATCC11539 |  |
| *Trametes versicolor* | NBRC4937 | TV1 |
| *T. versicolor* | NBRC30340 | TV2 |
| *Pycnoporus coccineus* | NBRC6489 | PC1 |
| *P. coccineus* | NBRC9495 | PC2 |
| *Trametes hirsuta* | NBRC7038 | TH1 |
| *T. hirsuta* | NBRC4917 | TH2 |
| *T. hirsuta* | NBRC6477 | TH3 |
| *Phanerochaete chrysosporium* | ME-446 |  |

Table S2 Oligonucleotides used as primers in this study.

| Primer name | Nucleotide sequence (5’-sequence-3’) |
| --- | --- |
| act-deg-F | AARGCNGGNTTYGCNGGNGAYGA |
| act-deg-R | RTTRTARAANGTRTGRTGCCA |
| act-F1 | CACAGTCGAAGCGTGGTATC |
| act-F2 | TATCCCATCGAGCACGGTAT |
| act-F3 | GTTACGATGGTGGTTTTCGG |
| act-F4 | TTCTTTTCTCCTTTCGCTGC |
| act-F5 | TGTACCATGTGTGGCAGAGC |
| act-F6 | GATGACGAAGTCGCTGCTCT |
| act-F7 | GGGGCGCGCCCTAAACGATATAATAA |
| act-R1 | ATGCAGCGAAAGGAGAAAAG |
| act-R2 | CGAACATGATCTGCGTCATC |
| act-R3 | GTGAGTAGGATGGGGTGCTC |
| act-R4 | CACTTACCTCCTTGCCGAAA |
| act-R5 | CCGGCGCGCCATCGTCACCTGAGA |
| gpd-deg-F | GTNGARWSNACNGGNGTNTTC |
| gpd-deg-R | CARTTNGTNGTRCANSWNGCRTT |
| gpd-F1 | CCAAGAAGGTCGTCATCACC |
| gpd-F2 | CCTATGTTCGTCGTGGGTGT |
| gpd-F3 | GTTTCCGTCGTTGACCTTGT |
| gpd-F4 | TCCCCTCAACAAGAATTTCG |
| gpd-F5 | AGTGAAAGCGAGTGCACAGA |
| gpd-F6 | CTTCCCTCACATCCCCTACA |
| gpd-F7 | GCGGCGCGCCCTAAATACAGACATTT |
| gpd-F8 | TCGCGGTTTGGAATTGTCTC |
| gpd-R1 | GCTGTTATTACACTTTCCTT |
| gpd-R2 | CATTGGAGACCTTCGCAGAC |
| gpd-R3 | ACACCCACGACGAACATAGG |
| gpd-R4 | ATGTGATCGCAGAGCAATGA |
| gpd-R5 | GGGCGCGCCGCCGCCCTGGCCGGCGTCCTT |
| gpd-R6 | GGAAGAGAGCGACATCGTTACTGAAGT |
| hph-F1 | CTGCGGCCGATCTTAGCCAGACG |
| hph-R1 | ACATTGTTGGAGCCGAAAT |
| lcc-F1 | ATCTTCGTGCTTTGCCTCAC |
| lcc-F2 | ACTTCAGTAACGATGTCGCTCTCTTCC |
| lcc-R1 | CCAGACCCTCTCTATGCTGG |
| lcc-R2 | TGGGCGCGCCTCAAAAGTCGTGCGACCCGT |
| lcc-R3 | GCTCAGCTGGACATTGGAG |

A/T/G/C;N, A/G;R, C/G;S, A/T; W, and C/T;Y

Fig. S1 Procedure for cloning genomic DNA fragments encoding *Gtact*.

The genomic DNA of *G. trabeum* cytosolic actin (*Gtact*) was cloned by a series of PCR procedures. The primers used are listed in Supplemental Table 2. The conserved region of cytosolic actin was amplified using degenerate forward primer act-deg-F1 and reverse primer act-deg-R1. The cDNA of KU-41 was used as template. The 3′-coding region was cloned by 3′-RACE using forward primer act-F1 and nested forward primer act-F2, respectively. Oligo dT-adaptor primer dT-RA was used for reverse transcription. Primer RA was used as reverse primer. Forward primer act-F1 and reverse primer act-R1 were used for amplification of genomic DNA to confirm the positions of the introns. The 5′- and 3′-untranslated regions of *Gtact* were cloned by inverse PCR procedure. Self-ligated genomic DNAs digested with *Apa* I, *Bam*H I, *Eco*R I, *Hin*d III, *Kpn* I, *Sac* I, and *Spe* I were used as templates. Forward primer act-F3 and reverse primer act-R2 were used for the first PCR amplification. The PCR product was diluted 100-fold and amplified using nested forward primer act-F4 and reverse primer act-R3. Forward primer act-F5 and reverse primer act-R4 were used to amplify the full length of the genomic DNA using error-prone PCR enzyme (PrimeSTAR HS). The PCR product was cloned in pCR4Blunt-TOPO, and sequenced using a GPS™-1 Genome Priming System kit. The plasmid harboring genomic DNA fragment of *Gtact* was designated p*Gtact*. Forward primer act-F6 and reverse primer act-R1 were used for RT-PCR to confirm the position of the introns.


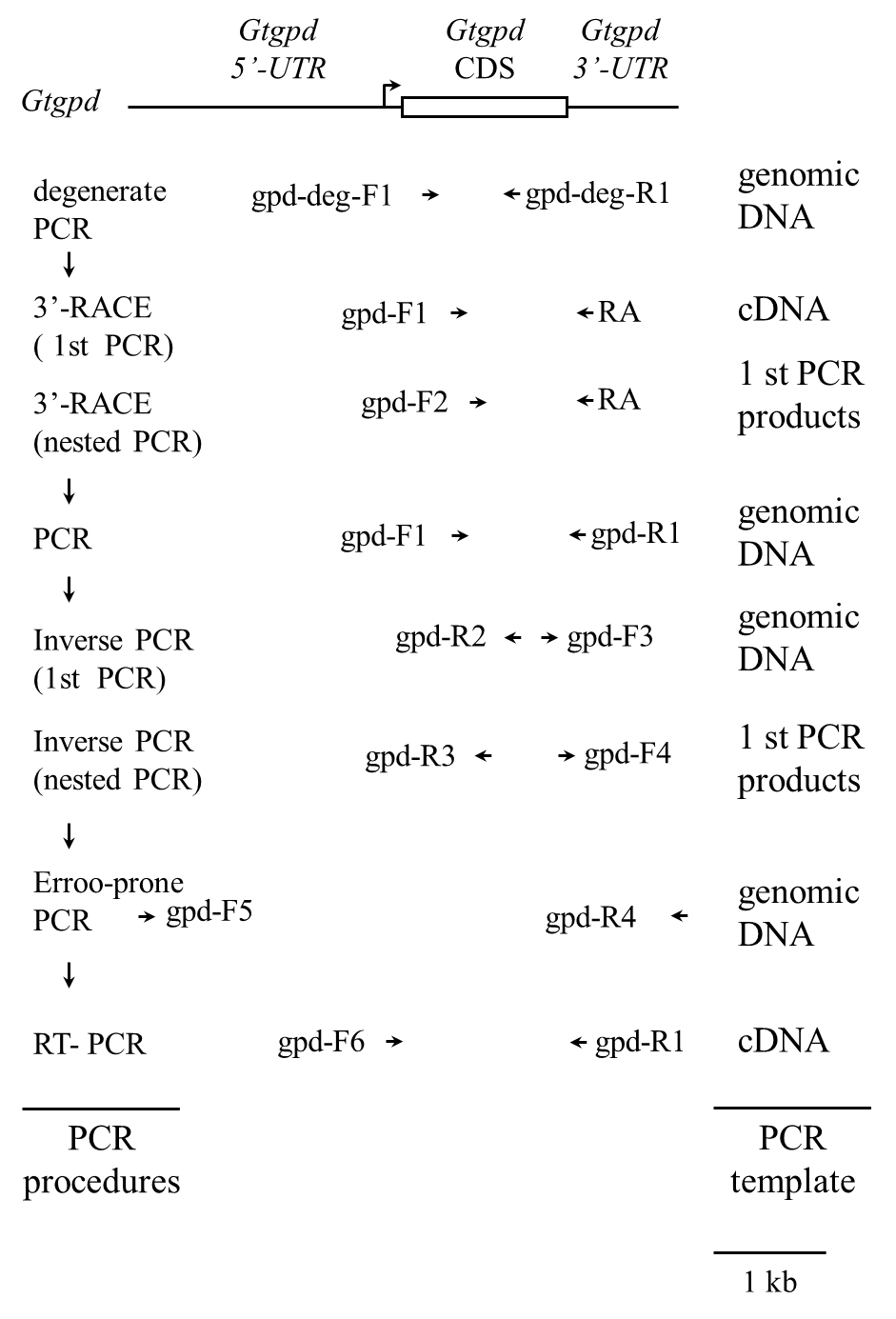


Fig. S2 Procedure for cloning genomic DNA fragments encoding *Gtgpd*.

The genomic DNA of *G. trabeum* glyceraldehyde-3-phosphate dehydrogenase (*Gtgpd*) was cloned by a series of PCR procedures. The conserved region of *Gtgpd* was amplified using degenerate forward primer gpd -deg-F1 and reverse primer gpd-deg-R1. The cDNA of KU-41 was used as template. The 3′-coding region was cloned by 3′-RACE using forward primer gpd-F1 and nested forward primer gpd-F2, respectively. Oligo dT-adaptor primer dT-RA was used for reverse transcription. Primer RA was used as reverse primer. Forward primer gpd-F1 and reverse primer gpd-R1 were used for amplification of genomic DNA to confirm the position of the introns. The 5′- and 3′-untranslated regions of *Gtgpd* were cloned by inverse PCR procedure. Self-ligated genomic DNAs digested with *Apa* I, *Bam*H I, *Eco*R I, *Hin*d III, *Kpn* I, *Sac* I, and *Spe* I were used as templates. Forward primer gpd-F3 and reverse primer gpd-R2 were used for the first PCR amplification. The PCR product was diluted 100-fold and amplified using nested forward primer gpd-F4 and reverse primer gpd-R3. Forward primer gpd-F5 and reverse primer gpd-R4 were used to amplify the full length of the genomic DNA using error-prone PCR enzyme (PrimeSTAR HS). The PCR product was cloned in pCR4Blunt-TOPO, and sequenced using a GPS™-1 Genome Priming System kit. The plasmid harboring genomic DNA fragment of *Gtgpd* was designated p*Gtgpd*. Forward primer gpd-F6 and reverse primer gpd-R1 were used for RT-PCR to confirm the position of the introns.


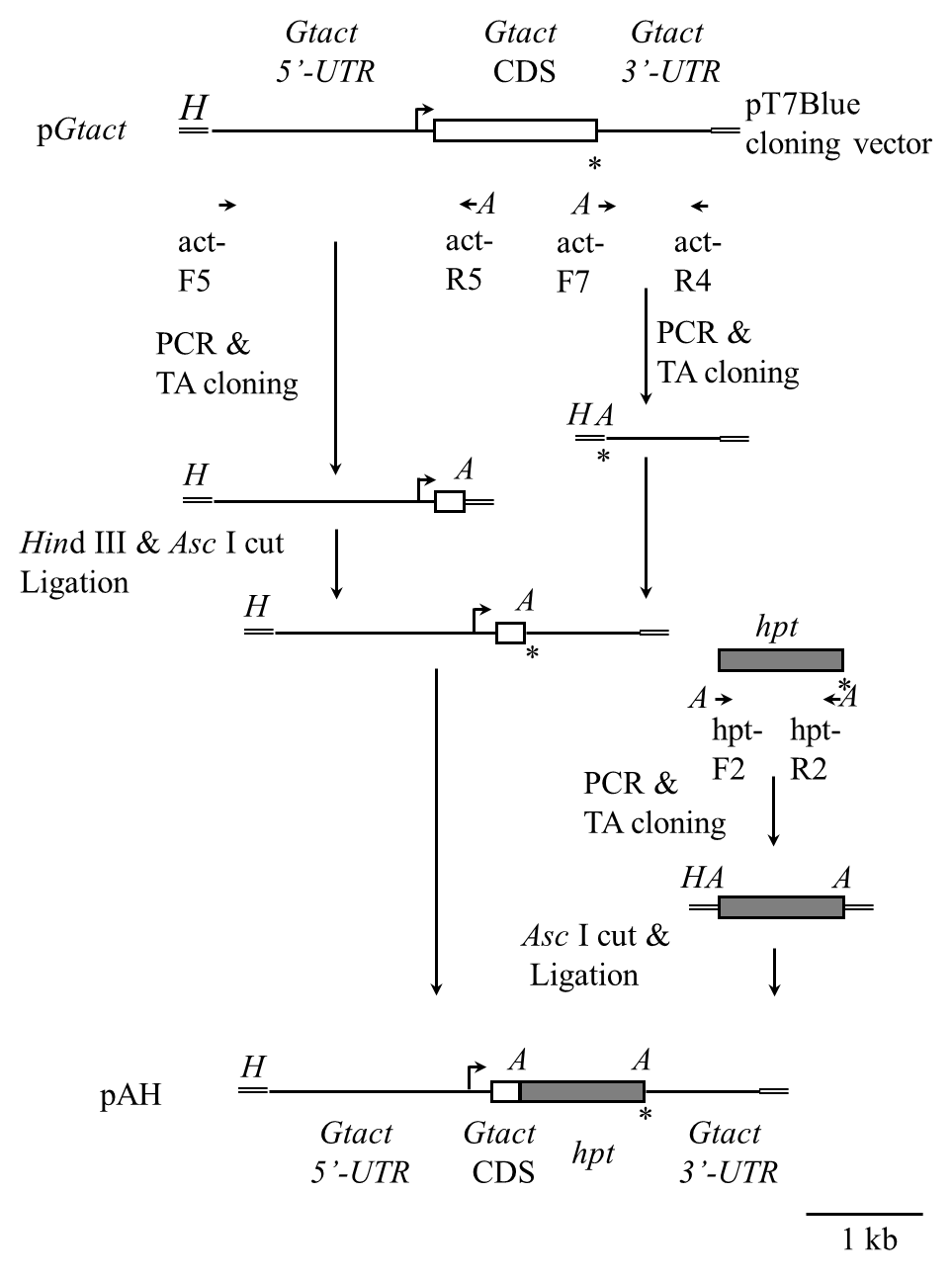


Fig. S3 Construction of marker plasmid pAH.

PCR mutagenesis procedures for addition of restriction enzyme sites (*Asc* I) were performed using Platinum Taq DNA Polymerase High Fidelity (Invitrogen). The PCR products were subcloned in pT7Blue T-vector (double line) and sequenced to ascertain the absence of PCR-induced error. A; *Asc* I, H; *Hin*d III, asterisk; stop codon.


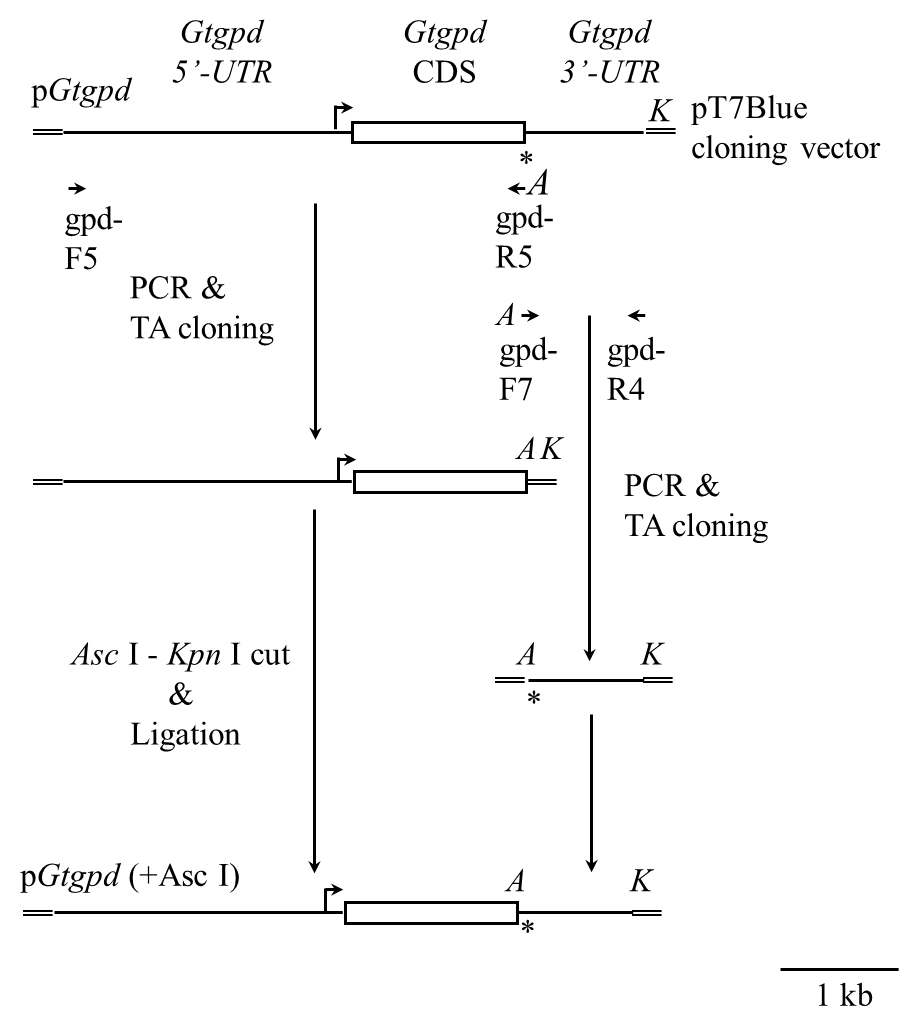


Fig. S4 Addition of an *Asc* I site in p*Gtgpd*.

PCR mutagenesis procedures for addition of restriction enzyme site (*Asc* I) were performed using Platinum Taq DNA Polymerase High Fidelity. *Kpn* I site is located in multicloning site of pT7Blue T-vector. The PCR products were subcloned in pT7Blue T-vector and sequenced to ascertain the absence of PCR-induced error. A; *Asc* I, H; *Hin*d III, asterisk; stop codon.


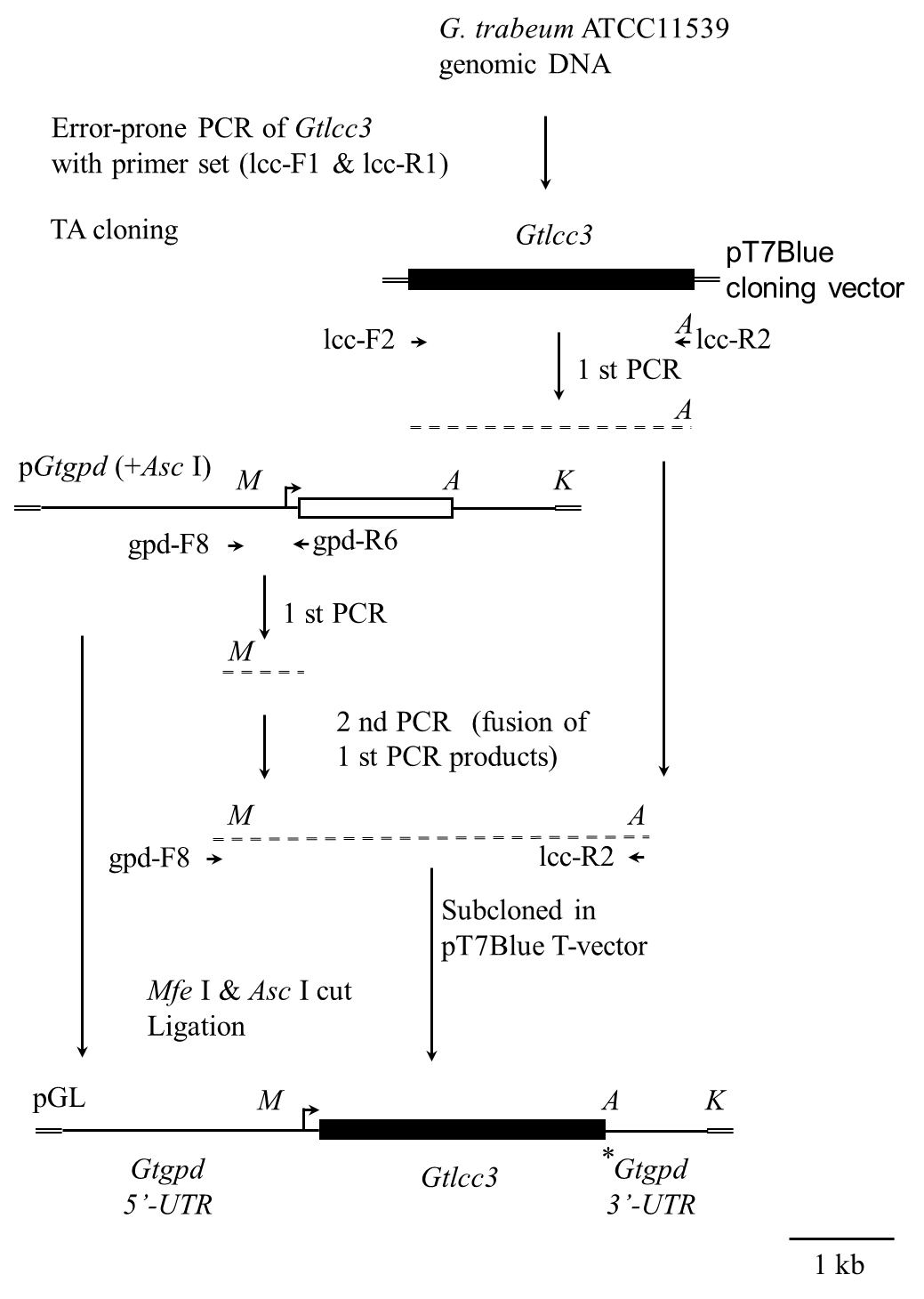


Fig. S5 Construction of pGL.

PCR mutagenesis procedures for addition of restriction enzyme site (*Asc* I) at the 3’ end of *Gtlcc3* and PCR-based fusion of PCR fragments were performed using Platinum Taq DNA Polymerase High Fidelity. The primer set lcc-F2 and lcc-R2 was used to prepare 1 st PCR product (*Gtlcc3*-*Asc* I site). The primer set gpd-F8 and gpd-R6 was used to prepare another 1 st PCR product. The 5’ end of primer lcc-F2 and gpd-R6 were designed to be complementary. The 1 st PCR products were mixed and used as PCR template to join *Gtgpd* 5’-untranslated region and *Gtlcc3* coding region in frame. The 2 nd PCR product was subcloned in pT7Blue T-vector and sequenced to ascertain the absence of PCR-induced error. The plasmid p*GtGPD* (+Asc I) and the 2 nd PCR product in the cloning vector were double-digested with *Mfe* I and *Asc* I and ligated to construct plasmid pGL. A; *Asc* I, M; *Mfe* I, H; *Hin*d III, asterisk; stop codon.

Fig. S6 Zymogram activity of the purified laccase enzyme with native PAGE (**1**:WT, **2**:L#61 G1, **3**:L#61 G5).

Activity staining of the laccase revealed a single protein band corresponded with activity of the laccase with 2,6-dimethoxyphenol.
